# Neurological recovery after ICH is mediated by the aryl hydrocarbon receptor-bilirubin interplay through improved erythrophagocytosis

**DOI:** 10.1177/0271678X251371375

**Published:** 2025-09-17

**Authors:** Xiurong Zhao, Shun-Ming Ting, Guanghua Sun, Jaroslaw Aronowski

**Affiliations:** Department of Neurology, McGovern Medical School, University of Texas Health Science Center at Houston, Houston, TX, USA

**Keywords:** Intracerebral hemorrhage, bilirubin, aryl hydrocarbon receptor, phagocytosis, cytoprotection

## Abstract

Hematoma clearance after ICH is a pro-hemostatic process aiming at repair/recovery and is achieved through microglia/macrophages (MMΦ)-mediated erythrophagocytosis. Upon the engulfment of masses of erythrocytes and toxic hemolysis products, hemoglobin and heme, phagocytes convert them to bilirubin (BrB). Bilirubin is essentially not soluble in water and when overproduced, it precipitates within the cell causing injury. Thus, keeping bilirubin soluble and at a low intracellular level is needed for proper function of MMΦ. Here, using cultured microglia (MG), we found that intracellular formation of BrB in microglia during erythrophagocytosis coincides with the activation of transcription factor AhR, and AhR target genes upregulation, including ligandin, a protein known for retention of BrB solubility, and Mrp1 known for mediating BrB efflux from the cell. Further studies showed that AhR contributed to MG’ self-protection from BrB toxicity for a more efficient phagocytosis. Using mouse ICH model, we established that AhR is abundant in MMΦ located near hematoma, and that AhR agonists, ITE, used as treatment for ICH, improved both hematoma clearance and neurological recovery. In support of important role of AhR in microglia in ICH, the selective AhR-deficiency in MG in mice worsened the hematoma clearance and impaired post-ICH recovery and weakened ITE from mediating therapeutic effect.

## Introduction

Intracerebral hemorrhage (ICH) is a devastating form of stroke with very high global morbidity and mortality, and carries the highest disability rate among survivors.^1^ Besides minimally invasive surgical hematoma evacuation for lobar hemorrhages, there is no approved effective treatment for the ICH.^2^ The benefit of hematoma evacuation is very revealing, as it provides direct evidence that hematoma is toxic to the brain and that the clearance of blood from the hematoma space is therapeutically beneficial.^3^

After ICH, immune cells, primarily microglia (MG) and bone marrow-derived macrophages (BMDM), together termed MMΦ, migrate to the sites of hematoma where they conduct phagocytosis/endocytosis-mediated engulfment of ICH-deposited cytotoxic RBCs and their lysis products, hemoglobin, heme, and iron. This process is a primary mechanism for the endogenous hematoma resolution.^4,5^ However, following the engulfment of the masses of RBC and their lysis product from hematoma, MMΦ must recruit various catabolic enzymes that, during the degradation process, generate toxic byproducts that may be damaging to MMΦ themselves.^6^ Thus, protecting MMΦ for sustaining their phagocytic and “reparative/trophic” functions is essential for effective hematoma resolution and post-ICH recovery.

Bilirubin (BrB) is a terminal product of the hemoglobin/heme degradation pathway in MMΦ, which is generated at high concentration after ICH, and it is synthetized by the rate-limiting enzyme, heme oxygenase-1 (HO-1), that is, robustly induced in response to ICH.^7–9^ Newly generated free BrB (fBrB) is almost insoluble in water.^10,11^ Without the presence of its binding proteins, fBrB aggregates at low concentrations (1 μM or even 140 nM).^12–14^ Its accumulation leads to the formation of metastable microsuspensions and coarser aggregates that precipitate on biological membranes and nucleic acid, causing structural and oxidative damage to intracellular organelles and DNA.^10,15^ Thus, the buildup of supraphysiological levels of fBrB in the MMΦ during their cleanup process after ICH could be toxic to the phagocytes and impede the cleanup process and, thus, recovery.

Intracellularly, BrB is normally bound to intracellular BrB-binding proteins (particularly ligandins, LGN)^16^ that help retain BrB solubility and facilitate its intracellular transport.^17–20^ LGN is the principal intracellular BrB-binding protein that, by binding to hydrophobic fBrB, increases its cytosol solubility^20,21^ and acts as an intracellular transporter,^22–24^ as does albumin in blood plasma.^25–27^ On the other hand, the unbound newly synthesized fBrB is normally exported out of the cell through BrB exporting proteins, including multidrug resistance-associated Protein 1 (Mrp1),^28–30^ which pumps fBrB out of the cells to reduce its intracellular level and prevent BrB aggregates formation.^31–33^ Interestingly, the expression of LGN and Mrp1 is achieved by activation of transcription factor aryl hydrocarbon receptor (AhR).^34,35^

AhR is a ligand-dependent transcription factor that binds to xenobiotic response element (XRE) in target gene promoters to regulate the expression of genes essential for metabolism/detoxification of pollutants, xenobiotics, and some endogenously produced metabolites.^36–38^ BrB is an endogenous AhR ligand.^39,40^

Here, we found that the intracellular BrB is increased in MG upon phagocytosis of RBC and that this coincides with AhR activation and the upregulation of AhR prototypic gene targets, including Mrp1 and LGN that could contribute to MG’ self-protection from fBrB toxicity for more efficient phagocytosis function. Also, we established that the AhR agonists used as treatment for ICH in mice improve hematoma resolution and neurological recovery after ICH and that the selective AhR deficiency in MG worsens the hematoma clearance process and impairs post-ICH recovery.

## Materials and methods

### Animals

All studies involved animal tissue followed the guidelines outlined in Guide for the Care and Use of Laboratory Animals from the National Institutes of Health and were approved by the Animal Welfare Committee of The University of Texas Health Science Center at Houston. All experiments used a randomization approach (coin toss; two coins were used if four animals were randomization), and analyses were conducted by investigators blinded to treatment assignments (animals were coded for group allocation) following STAIR recommendations^41^ and ARRIVE guidelines.^42^ Animals were fed a standard mouse/rat diet and housed in standard mouse/rat cages on a 12-h inverted light–dark cycle. Behavioral analyses were carried out from the hours of 10:00 AM to 4:00 PM.

TMEM-AhR-KO mice were produced in our animal facility by crossing Tmem119-CreERT2 transgenic mice (C57BL/6-*Tmem119^em1(cre/ERT2)Gfng^*/J, Stock No. 031820, F/M) and AhR^flox^ mice (F/M, Ahr^tm3.1Bra^/J, Stock No. 006203) for two generations to produce Tmem119-Cre^+^-AhR^loxP^. By crossing Tmem119-Cre^+^-AhR^loxP^ (MG-AhR-KO) and AhR^flox^ mice, we produced the progeny with or without Cre expression, which provided either Tmem119-Cre^+^-AhR^loxP^ (MG-AhR-KO mice) or Tmem119-Cre^−^-AhR^loxP^ (the control mice) for our study. For genotyping, we used the PCR method provided by the Jackson Lab. The following PCR primers were used for the detection of the recombined and non-recombined AhR allele, respectively: primer oIMR6075 (5′-CAG-TGG GAA-TAA-GGC-AAG-AGT-GA) and oIMR6076 (5′-GGT-ACA-AGT-GCA-CAT-GCC-TGC), producing mutant band at 140 bp and WT band at 106 bp. The TMEM-Cre PCR used 16,504 (5′-ATC-GCA-TTC-CTT-GCA-AAA-GT), 42,648 (CAG-TAT-GTG-GGG-TCA-CTG-AAG-A), and 42,649 (5′-ACT-TGG-GGA-GAT-GTT-TCC-TG), producing 280 bp mutant band and 378 bp wild-type band. To measure Cre and AhR mRNA expression in the TMEM-AhR-KO mice or MG, we used primers (Cre: GCATTACCGGTCGATGCAACGAGTG and GAACGCTAGAGCCTGTTTTGCACGTTC; AhR: TCTAAGCGACACAGAACCG and GCTGACGCTGAG CCTAAGA). All the experimental groups of mice were age- and sex-matched. Throughout the study, we used 4–6-month–old male and female mice.

### Phenotypic analysis

The MG-AhR-KO mice are normal regarding food intake, body weight growth and blood glucose. For measurement of mouse body weight and peripheral white-blood-cell (WBC), data were collected and compared versus control littermates. Blood glucose was established on whole venous blood using an automatic glucometer (One Touch Basic, Lifescan). Total peripheral blood WBC was counted by lysing 10 µl fresh tail blood in 400 µl of 2% acetic acid and counted under 10× phase-contrast microscopy using hemocytometer.

### ICH model in rodents

The ICH model in mouse was based on intra-striatal injection of autologous blood, as we described.^4,43,44^ Briefly, male/female mice (25–30 g) under isoflurane anesthesia (delivered by the face mask in a 1:1 mixture of oxygen:nitrous oxide) were immobilized onto a stereotaxic frame. A one-mm-diameter burr hole was drilled in the skull and a 31-gauge stainless steel cannula was inserted for the blood infusion (15 µl/mouse) at a speed of 5 µl/min. The core body temperature was maintained at 37°C ± 0.5 °Cduring surgery and during the first hour after the surgery. We experienced no mortality after ICH in this study.

### Animal perfusion and tissue collection

All animals were fatally anesthetized with chloral hydrate (0.5 g/kg; i.p.) and intracardially perfused with ice-cold PBS. For histology or biochemical analyses, the brains or the sub-dissected brain tissues representing hematoma-affected striatum were snap frozen in −80 °C 2-methylbutane and stored in −80 °C prior to cryosectioning, RNA isolation, or protein extraction.

### Neurological deficits score (NDS)

All behavioral tests in mice were conducted in a quiet and low-lit room by an experimenter blinded with respect to the treatment assignment. Pre-tests were done to potentially exclude abnormally behaving animals. The NDS was determined by a battery of behavioral tests, including Foot-fault, Postural Flexing, and Corner turning test, which were used to measure the severity of the neurological deficits, as we reported earlier.^4,45^ Higher NDS values indicate more severe neurological deficits.

### Hematoma size measurement

Hematoma resolution was assessed by measuring the amount of hemoglobin (Hb) remaining in the hematoma-affected brain on day 7 after ICH, as described.^4^ Briefly, mice under deep chloral hydrate anesthesia were perfused with ice-cold PBS to remove intravascular blood. Intraparenchymal Hb in the homogenized ipsilateral striatum, dissected from coronal brain cryosections, was measured using Drabkin’s reagent and the peripheral blood was used as standard.^4,46^ The data were expressed as blood volume per brain homogenate.

*Drug/reagents administration:* The pharmacologic reagents used here, including ITE, TMF, CH-223191, tamoxifen, and SnPP, were pre-tested for their dose-response in microglia cultures regarding their toxicity to microglia using MTT assay and morphological observation (data not included). The doses employed here are not toxic, and similar to the doses used by the others.^47–51^

*Bilirubin* (BrB, B4126, Sigma–Aldrich) was first dissolved in 0.1 N NaOH at 50 mg/ml, then add 5.6 mM sodium taurocholate to get 5.85 mg/ml (10 mM), then 1:1 diluted in an isotonic solution containing 0.5% Na_2_CO_3_ and 0.52% NaCl, which was used for the cell culture. The final concentration of BrB in the culture experiment is 10 µM. The solvent used for dissolving BrB was used as the vehicle control.

*Tamoxifen* (TAM, Sigma, T5648) was delivered i.p. at 3 mg/day/mouse (dissolve in corn oil at 20 mg/ml) on day 1 and then 1 mg/day on days 3 and 5 to activate Cre expression; and then wait for 1 week after the last dose, before the mouse was subjected to ICH.

*ITE* (R&D Systems), AhR agonist,^47,48^ was dissolved in 50% DMSO at 20 mg/ml and 1:9 diluted with saline. ITE treatment for mice was given at 8 mg/kg in 100 µl, i.p., starting 24 h after ICH and then daily for 5 days. Five percent DMSO in saline was used as the vehicle control in the animal experiment. For the cell culture experiment, the concentration of ITE in the culture medium was 2 µM. The DMSO in culture medium (0.001%) was used as vehicle control.

*6, 2′, 4′-trimethoxyflavone* (TMF, T4080, Sigma-Aldrich) was dissolved in DMSO at 5 mg/ml (16 mM). TMF was used at the final concentration of 2 µM in the cell culture experiment. The DMSO in culture medium (0.0125%) was used as vehicle control.

*CH-223191* (C8124, Sigma-Aldrich) was first dissolved in DMSO at 10 mg/ml to produce 30 mM stock. The final concentration of CH-223191 in the culture medium was 5 µM. DMSO in culture medium (0.0167%) was used as vehicle control.

*Tin protoporphyrin IX* (SnPP, S7879, Sekkeckchem.com) was solubilized in a 1:1 95% ethanol/0.1 N NaOH mixture (v/v) at 20 mM and used at 10 µM in the cell culture medium. The solvent for SnPP (1:1 of 95% ethanol:0.1 N NaOH mixture) was used as vehicle control.

### Primary brain glial culture and microglia isolation

The cortices of postnatal 1–2-day-old Sprague–Dawley rat, C57/BJ6 Mice (WT), TMEM-AhR-KO or AhR^loxP^ mice pups were dissected and dissociated by trituration as described previously.^4^ The dissociated cells were plated in 75 cm^2^ tissue culture flasks in DMEM with 10% fetal bovine serum, and maintained in a CO_2_ incubator (5% CO_2_, 21% O_2_) at 37.0 °C ± 0.5 °C. Half of the culture medium was changed every 3 days. After a total of 12–15 days in culture, the astrocytes form a confluent layer signifying that the cultures are ready for microglia isolation. The suspended or the loosely adherent MG were harvested by shaking at 220 rpm/5 min. The detached MG were collected and re-plated onto poly-D-Lysine-coated TC plates with or without German-glass inserts at a density of 1–4 × 10^5^ cells/ml. In the cultures prepared from TMEM-AhR-KO mice, to induce Cre expression, we added 0.02 mg/ml of 4-hydroxytamoxifen (4-OH-TAM, SML1666, Sigma-Aldrich, Stock: 5 mg/ml in methanol) into the cultures, which was maintained in the cultures for 48 h prior to harvesting microglia. The concentration of methanol and corn coil in the culture medium is 0.4% and 1% in PBS, respectively, which was used as the vehicle control.

### Immunofluorescence (IF)

The IF was performed as we described.^4^ Briefly, the frozen brain coronal sections (20 µm) or cultured cells on German Glass were treated with 2% formalin for 15 min and incubated in the primary antibodies overnight at 4 °C (antibodies are listed in Table 1). The relative secondary antibodies conjugated with IgG-Alexa Fluor 488, 546, or 647 (Invitrogen, USA) was used to visualize the signals.

**Table 1.** PCR primers and antibody used in this study.

PCR primer list						
Gene name	Primer sequence		Product size (bp)	Gene code	Primer location	Used in figure
Rat GAPDH	R-GAPDH-F	ACCCAGAAGACTGTGGATGG	81	NM_008084.3	784	Figure 2(c)
	R-GAPDH-B	GGATGCAGGGATGATGTTCT			864	
Mouse GAPDH	M-GAPDH-F	TGTTCCTACCCCCAATGTGT	396	NM_001001303	754–	Figure 4(b)
	M-GAPDH-B	TGTGAGGGAGATGCTCAGTG			–1149	
Mouse CD68	M-CD68-F	CCAATTCAGGGTGGAAGAAA	328	NM_009853	457–	Figure 3(e)
	M-CD68-B	TTGCATTTCCACAGCAGAAG			–784	
Rat HO-1	R-HO-1-F	TGGGTCCTCACTCTCAGCTT	382	X13356	885–	Figure 2(c)
	R-HO-1-B	GTCGTGGTCAGTCAACATGG			–1266	
Cre	Cre-F1	GCATTACCGGTCGATGCAACGAGTG	374	AF234173.1	472–496	Figure 3(e)
	Cre-B1	GAACGCTAGAGCCTGTTTTGCACGTTC			820–846	
Mouse AhR-KO	M-AhR-KO-F1	CGGTGCAGAAAACAGTAAAGC	190	NM_013464	Exon 1	Figure 3(e)
	M-AhR-KO-B2	GCTGACGCTGAGCCTAAGA			Exon 2	
Rat AHR	R-AHR-F1	GTCCTCAGCAGGAACGAAAG	454	NM_013149	2040–	Figure 2(c)
	R-AHR-B1	TCTGCCGAGTAGGCTTCATT			–2493	
Mouse AHR	M-AHR-F1	AGCATGCAGAACGAGGAGTT	417	NM_013464	2005–	Figure 5(a)
	M-AHR-B1	CTGAGCAGTCCCCTGTAAGC			–2421	
Rat Cyp1a1	R-Cyp1a1-F	CAAAATACTGGCACGGAGGT	374	NM_012540	2002–	Figure 2(c)
	R-Cyp1a1-B	AGCGGTTCATGACTGTACCC			–2375	
Rat Cyp1b1	R-Cyp1b1-F	GAGCTCGCTGTCTACCCAAC	380	NM_012940	410–	Figure 2(c)
	R-Cyp1b1-B	GCTCTGAGTAGTGGCCGAAC			–789	
Mouse Cyp1a1	M-Cyp1a1-F	CAGACCTCAGCTGCCCTATC	457	NM_009992	1275–	Figure 5(a)
	M-Cyp1a1-B	AGAAGACCGCATCTGCACTT			–1731	
Mouse Cyp1b1	M-Cyp1b1-F	CAGCAACTTCGTTCTGGACA	387	NM_009994	1180–	Figure 5(a)
	M-Cyp1b1-B	ACAGGCAAAAAGCTGGAGAA			–1566	
Rat LGN	R-GSTa2-F	CGCCACCAAATATGACCTCT	375	NM_017013	288–	Figure 2(c)
	R-GSTa2-B	GGCTGCAGGAACTTCTTCAC			–662	
Mouse LGN	M-LGN-F	GGTAGAGATCGACGGGATGA	320	M73483.1	197–	Figures 4(b) and 5(a)
	M-LGN-B	GGAGTTCAACCAGGGCAATA			–518	
Rat ARNT	R-ARNT-F	CTTGGCTCTGTGAAGGAAGG	300	AY264361	885–	Figure 2(c)
	R-ARNT-B	TAAGAGCTCCTGTGGCTGGT			–1184	
Rat Mrp1	R-Mrp1-F	GACAGACGCAGTAGGGAAGC	391	NM_022281	2748–	Figure 2(c)
	R-Mrp1-B	GCCAAATACTGCCACACCTT			–3138	
Mouse Mrp1	M-Mrp1-F	GGCAGTGGAGAGACTGAAGG	475	NM_008576	3806–	Figures 4(b) and 5(a)
	M-Mrp1-B	GTCAGGCAAAGCTGACACAA			–4280	
Mouse 18S	M-18S-F	AAACGGCTACCACATCCAAG	479	NR_003278	448–	
	M-18S-B	CCCTCTTAATCATGGCCTCA			–926	
Mouse TNFα	M-TNFα-F	CCACATCTCCCTCCAGAAAA	702	NM_013693	131–	
	M-TNFα-B	CGGACTCCGCAAAGTCTAAG			–832	
Mouse IL-1β	M-IL-1β-F	GGGCCTCAAAGGAAAGAATC	612	NM_008361	599–	
	M-IL-1β-B	CTCAGTGCAGGCTATGACCA			–1210	
Mouse TGFβ	M-TGFβ-F	TGAGTGGCTGTCTTTTGACG	456	NM_011577	1446–	
	M-TGFβ-B	TGGTTGTAGAGGGCAAGGAC			–1901	
Mouse Arginase 1	M-Arg1-F	AAGCTGGTCTGCTGGAAAAA	310	NM_007482	200–	
	M-Arg1-B	CTGGTTGTCAGGGGAGTGTT			–509	
Mouse COX2	M-COX2-F	TCCTCCTGGAACATGGACTC	321	NM_011198	1296–	
	M-COX2-B	CCCCAAAGATAGCATCTGGA			–1616	
Antibody list						
Ab name	Cat no.	Company	Use			
CD11b	MCA711	Serotec	IF			
CD68	MCA341R	Serotec	IF			
AhR	NB100-2289	Novus Biologicals	IF			
AhR	NB100-128	Novus Biologicals	WB, IF, and EMSA, neutralizing			
LGN (GSTa2)	PA5-100255	ThermoFisher	IF and WB			
Mrp1	sc-53130	Santa Cruz	WB			
Mrp1	PA5-88082	Invitrogen	IF			
Rat RBC	20R-RR012	Fitzgerald	IF for rat RBC			
Mouse RBC	20R-RR009	Fitzgerald	IF for mouse RBC			
GAPDH	AB2302	Millipore	WB			

### Double or triple IF and cell counting

was used to localize AhR, Mrp1, LGN, or RBC to a specific cell type (CD11b- or CD68-labeled MG). DAPI labeled the nuclei.

For AhR, CD11b double-positive cell-quantitation in brain sections after ICH, the object-based colocalization analysis was performed using ImageJ. Briefly, a region of interest (ROI) outlining CD11b positive signal was generated from IF and then the number of nuclei (DAPI) within CD11b^+^ ROI was identified. Since AhR localize and also translocate into the nucleus upon activation, we quantitated the percentage of nuclei that contains AhR signals (*N* = 5 animals).

### Image capture

A Zeiss Axioskop 2 microscope equipped with CCD camera and operated by MetaMorph 6.2 software, or a Zeiss Confocal microscopy LSM 800 operated by Zen software was used for image acquisition. The fluorescence labeled cells were visualized at Ex/Em of 490/520 for Alexa 488, Ex/Em of 550/575 nm for Alexa 546, Ex/Em of 650/675 nm for Alexa Fluor 647, and Ex/Em of 365/480 nm for DAPI.

### MTT assay

The cell injury was assessed with MTT assay, using CytoTox 96 Non-Radioactive Cell Proliferation Assay kit (Promega). The assessments were performed according to the manufacturer’s instruction. Briefly, 2 × 10^5^ MG/ml were plated in a 96-well plate. After exposure of MG to RBC for 4 h, the dye solution was added into each well and the cells were incubated for 2 h in a CO_2_ incubator at 37.0°C ± 0.5 °C. After adding the stop solution, the absorbance (OD) was recorded at 570 nm.

### RNA isolation and RT-qPCR

Total RNA from harvested cells or tissues were isolated using Trizol reagents. Complementary DNA (cDNA) was synthesized from 1 µg of RNA using amfiRivert Platinum One (GenDEPOT) following manufacturer’s protocol. The mRNA level analysis was performed on a Mastercycler realplex (Eppendorf) with SYBR Green-based real-time PCR system (Thermo Fisher Scientific) or regular PCR primer sets (the primers used for the PCR are listed in Table 1). The glyceraldehyde-3-dehydrogenase (GAPDH) or 18S was used as an internal control. For expression quantification, Real-Time PCR data was calculated using the delta–delta Ct method and expressed as Log_2_FC.

### Intracellular BrB assay

The total and direct BrB amount in the MG-lysates were measured using a Bilirubin Assay Kit (MAK126, Millipore–Sigma). Briefly, the MG were lysed in 0.1 N NaOH with 5.6 mM sodium taurocholate (2 × 10^4^ cells/50 µl), then 1:1 diluted in an isotonic solution containing 0.5% Na_2_CO_3_ and 0.5% NaCl. After a centrifugation at 1000*g* for 1 min, the supernatant was directly used in the assay following the manufacture’s instruction. The total BrB in the cell-lysate was expressed as µg/ml.

### Electrophoretic mobility shift assay (EMSA) for AhR

The activity of AhR was assessed by using AhR EMSA kit (Signosis, Inc.). Briefly, the nuclear extract from MG was produced using Nuclear Extraction kit (Signosis, Inc.) followed by incubating nuclear extract with AhR DNA probe. The resulting protein:DNA complexes were separated using a non-denaturing polyacrylamide gel. After electrophoretic transfer onto a nylon membrane, the amount of probe was quantified using Streptavidin–HRP and chemiluminescent substrate. Optical density was determined using Kodak Analysis (EDAS) 290 system.

### Phagocytosis assay for RBC

Peripheral blood RBC were isolated using column density gradient centrifugation (BD Vacutainer^®^ CPT™; BD Biosciences) as described.^4^ MG’ erythrophagocytosis activity in vitro was performed as described.^4^ Briefly, the carboxyfluorescein diacetate succinimidyl ester (CFDA)-labeled RBCs were added to the cultured MG and incubated for the indicated duration. The phagocytosis was stopped by removing the culture medium that contains the remaining/not yet phagocytosed RBCs. After washing with PBS, the MG were lysed in ddH_2_O and the CFDA in MG was quantified at 490/520 nm on a fluorescence plate reader and used as an index of phagocytosis. The RBC used for phagocytosis assay were from the same animal species but not from the same animals for producing microglia.

### Small interfering RNA transfection

siRNA probes targeting mouse AhR (sc-29655), LGN (sc-44628), Mrp1 (sc-35961, SC), and the scrambled control siRNA (sc-37007) were from Santa Cruz. Primary mouse MG were seeded on 24-well plates (3 × 10^5^ cell/ml) and transfected with each siRNA (10 nmol/l) using siRNA transfection reagent (sc-29528) for 36 h following the manufacturer’s instructions, prior to testing MG in phagocytosis assay. The knockdown efficacy of AhR or Mrp1/LGN were confirmed by checking the expression of AhR signature genes with RT-PCR, or cells’ phagocytosis function, respectively.

### Western blot

Protein samples were prepared from mouse MG lysates, as described.^4,43^ Briefly, protein samples were separated on 4%–20% gradient tris-glycine SDS gels (EC6021, Thermo Fisher Scientific) followed by electrophoretic transfer onto nitrocellulose membranes. Antibodies for LGN (PA5-100255, ThermoFisher), Mrp1 (sc-53130, Santa Cruz), and GAPDH (AB2302, Millipore) were used for immunoblotting. Horseradish peroxidase (HRP)-conjugated immunoglobulin G (IgG) and an enhanced chemiluminescent substrate (Pierce ECL; Thermo Fisher Scientific) were used to visualize protein bands on X-ray films. The optical density (OD) was determined using Kodak Analysis (EDAS) 290 system and normalized by intensity of control band (GAPDH).

### Statistical analysis

All statistical analyses, including correlation analysis were performed using GraphPad Prism 7. Two-way analysis of variance (ANOVA) was used to assess data with two grouping variables. Remaining data were analyzed using one-way ANOVA. The Tukey test was used for pairwise comparisons. Non-paired *t*-test was used when two groups were compared. All data are presented as mean ± SD.

## Results

### Engulfment of RBC by MG generates free bilirubin and activates AhR

**Figure 1.**
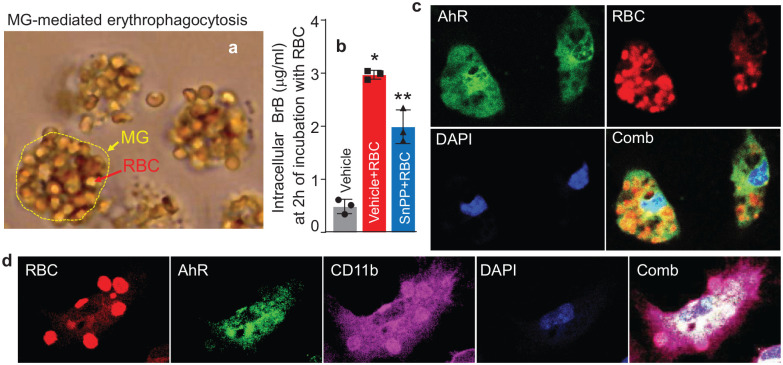
(a) Phase-contrast micrograph of mouse microglia (MG; outlined in yellow) in culture at 6 h after exposure to RBC. (b) Total intracellular BrB (in the cell lysate) at 6 h after exposure MG to RBC with or without 10 µM SnPP. Data are mean ± SD (*n* = 5), analyzed by one-way ANOVA. *p ⩽ 0.05 compared with the vehicle control. **p ⩽ 0.05 versus all other groups. (c) Double immunofluorescence of AhR and RBC, and (d) triple immunofluorescence of AhR, RBC, and CD11b to demonstrate the colocalization of AhR in mouse MG upon phagocytosis of RBC. The goat anti-AhR-Alexa Fluor 488 (green), rabbit anti-mouse RBC-Alexa Fluor 546 (red), and rat anti-CD11b-Alexa Fluor 647 (purple) were used to visualize the immunofluorescence signals in mouse MG, 6 h after exposure to RBC.

It is well-established that upon engulfment of RBC or Hb, phagocytes utilize inducible HO-1 to enzymatically degrade heme derived from Hb/RBC to generate CO, iron, and biliverdin.^52^ Biliverdin, consequently, is converted to BrB. We earlier reported that MG in culture are capable of phagocytosing RBC^4^ and here Figure 1(a). Here, we found that MG in culture, upon phagocytosis of RBC, significantly elevated the intracellular levels of BrB, which can be inhibited by the HO-1 inhibitor, SnPP (Figure 1(b)).

**Figure 2.**
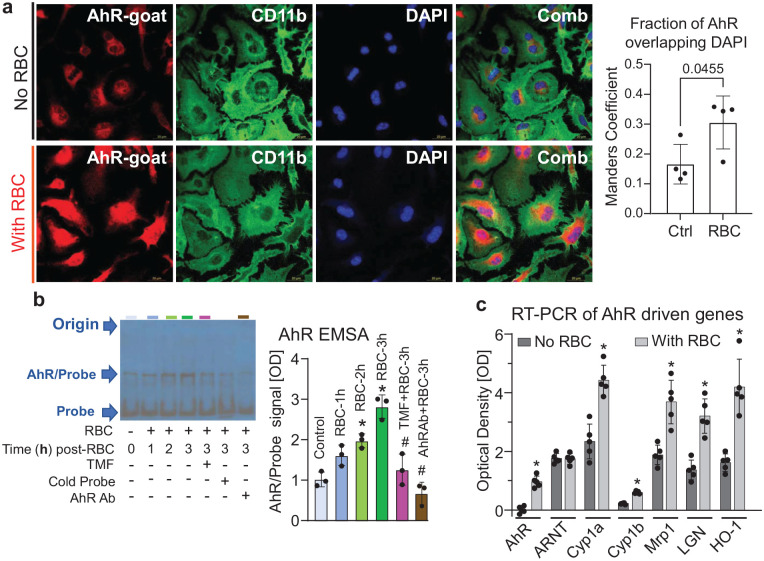
(a) Triple immunofluorescence staining of AhR (red), CD11b (green), and nucleus (DAPI) in cultured mouse MG at 2 h after exposure to RBC (bottom row) versus saline (no RBC) control. Note increased intranuclear presence of AhR in the MG exposed to RBC. The bar graph showing colocalization analysis performed using ImageJ JACoP plugin. The fraction of AhR pixels showing overlap with DAPI pixels, in four different MG culture images, was assessed as Manders’ coefficient. *p* = 0.0455, unpaired *t*-test. (b) AhR EMSA image in rat MG and a bar graph to demonstrate activation of AhR at indicated time (0–3 h) after exposure of MG to RBC with or without 2 µM TMF or 20 ng of AhR neutralizing antibody. (c) Bar graph to demonstrate activation of AhR based on upregulation of AhR-regulated genes in rat MG upon exposed to RBC. The gene expression was measured by RT-PCR. The data was first normalized by the internal control (GAPDH) and then the ODs were presented as mean ± SD (*n* = 5) and analyzed by paired *t*-test. *p ⩽ 0.05 compared to the control (no RBC). OD: optical density.

While BrB have numerous biological and physical effects on the phagocytic cells’ function and integrity, one intracellular role of BrB is to directly activate the transcription factor AhR.^39,40,53^ Thus, we next, using primary MG in culture and immunofluorescence, established that MG indeed express AhR (Figure 1(c)) and that the exposure of MG to RBCs leads to activation of AhR, as demonstrated by both the AhR immunofluorescence showing RBC-induced nuclear translocation of AhR (Figure 2(a)), and by electrophoretic mobility shift assay for AhR (EMSA; Figure 2(b)). The effect of RBC on AhR activation characterized with EMSA shows that the activation of AhR is a time-dependent process, which can be significantly reversed by either AhR inhibitor, 6,2′,4′-trimethoxyflavone (TMF) or an AhR neutralizing Ab (NB100-128; Novus; Figure 2(b)). Finally, using qPCR to assess mRNA expression of MG in response to RBC, we demonstrated a robust amplification of prototypic AhR target genes, such as *Cyp1a1*, *Mrp1*, and *Gsta2* (ligandin; LGN; Figure 2(c)), further documenting that AhR is activated in MG during the erythrophagocytosis.

### AhR activation in MG during erythrophagocytosis contributes to increased phagocytic functions

**Figure 3.**
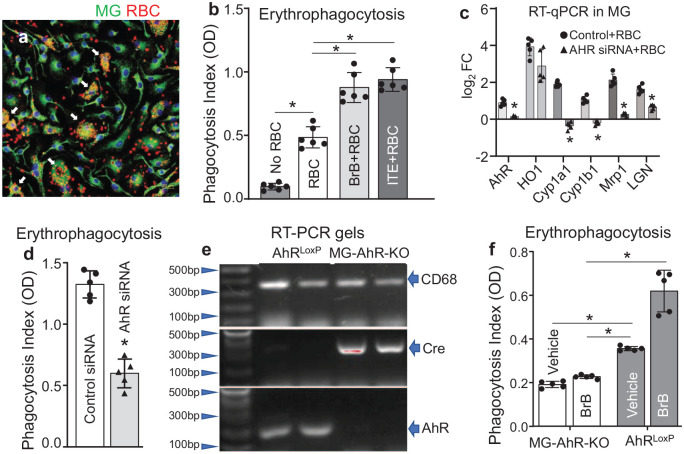
(a) Immunofluorescence of CD68 (green) and RBC (red) in cultured rat MG at 2 h after exposure to RBC showing representative image of RBC being internalized by the control MG. White arrows indicate erythrophagocytosis by the individual MG. DAPI was used to label the nuclei (blue). Bar = 20 µm. (b) Phagocytosis Index in rat MG at 2 h after exposure to RBC. Primary rat brain MG were pre-incubated with 10 µM BrB or 5 µM ITE (AhR agonist) for 16 h. CFDA-labeled RBCs were added to the MG culture at a ratio of 20:1 (RBC:MG) and incubated for 2 h. The non-phagocytosed by MG RBCs (floating in the medium) were aspirated, and the MG were washed with PBS and harvested for fluorescence intensity (OD) reading at (490/520 nm) as Phagocytosis Index. The data are mean ± SD (*n* = 6) and analyzed by one-way ANOVA. *p ⩽ 0.05. (c) Bar graph of qRT-PCR of prototypic AhR-regulated genes in mouse MG exposed to RBC in presence or absence of AhR siRNA. The data are shown as folds difference (Log_2_FC) over the no-RBC control in AhR- or control-siRNA-treated MG. Data are mean ± SD (*n* = 5) and analyzed by paired *t*-test. *p ⩽ 0.05. (d) Phagocytosis Index in mouse MG at 6 h after exposure to RBC following the knockdown of AhR with siRNA. Primary mouse MG in culture were incubated with AhR-siRNA or the control-siRNA at 10 nmol/l for 36 h, followed by adding CFDA-labeled RBC at a ratio of 20:1 (RBC:MG). The non-phagocytosed RBC (floating in the medium) were aspirated, and the MG were washed with PBS and harvested for the assessment of RNA expression and phagocytosis assay. Data are mean ± SD (*n* = 5) and analyzed by paired *t*-test. *p ⩽ 0.05. (e) Representative RT-PCR gels showing Cre expression in MG from MG-AhR-KO but not loxP control mice and corresponding AhR expression reduction in the cultured MG-AhR-KO MG. (f) Phagocytosis Index of mouse MG cultured from MG-AhR-KO and AhR^loxP^ mice at 6 h after exposure to RBC. The cultured MG were exposed to CFDA–RBC at a ratio of 1:20 and incubated for 6 h. Ten uM BrB was added to the MG 16 h prior to adding RBC. The data are mean ± SD (*n* = 5) and analyzed by one-way ANOVA and followed by Tukey’s multiple comparison test. *p ⩽ 0.05.

Since AhR is activated in MG upon engulfing RBC (Figure 3(a)), we next tested if AhR activation influences phagocytosis. Using primary MG in culture and 1′H-indole-3′-carbonyl)-thiazole-4-carboxylic acid methyl ester (ITE) to activate AhR,^47,48^ we established that 2 µM ITE nearly doubled the phagocytic index of MG (Figure 3(b)). Knowing that BrB can activate AhR, we pre-treated MG with BrB prior to exposing them to RBC and found that BrB could significantly enhance it to a similar extent as ITE does (Figure 3(b)).

### AhR knockdown in MG reduces the expression of AhR-regulated genes and inhibits erythrophagocytosis

Since the activation of AhR stimulates erythrophagocytosis, we next tested if the loss of AhR could impair this process. Thus, we incubated MG with AhR-siRNA (vs scrambled RNA) for 36 h prior to adding RBC. We validated that the siRNA effectively reduced the expression of AhR-regulating genes in MG (Figure 3(c)). Finally, we established that AhR-siRNA significantly reduced the erythrophagocytosis by MG (Figure 3(d)). To further document the causal role of MG’ AhR in erythrophagocytosis, we performed the same assay using the MG generated from MG-specific AhR knockout mice (MG-AhR-KO; generated by crossing AhR^loxP^ mice with TMEM119-cre mice). Similar to AhR-siRNA, the AhR-KO MG also showed a robustly reduced erythrophagocytosis, as compared to the control (AhR^loxP^) MG (Figure 3(f)). Important for establishing causality for BrB acting as activator of AhR needed for erythrophagocytosis, treatment of the control MG with 10 µM BrB improved the erythrophagocytosis by 73.3% in the AhR^loxP^ control MG, but only by 18.8% in the AhR-KO MG.

### AhR gene targets, ligandin and Mrp1, enhance MG integrity during erythrophagocytosis

**Figure 4.**
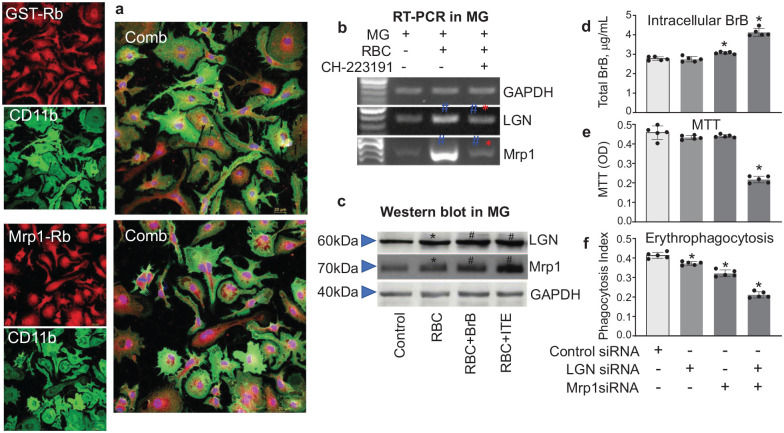
(a) Immunofluorescence of Mrp1 (red) or LGN (red) in primary mouse MG cultures. The MG (green) was labeled for CD11b. DAPI was used for the labeling nuclei (blue). Bar = 20 µm. (b) RT-PCR of Mrp1 and LGN expression in WT mouse microglia. The expressions of Mrp1 and LGN are induced by RBC (6 h after exposure to RBC and inhibited by AhR antagonist (10 µM CH-223191). The OD of each protein was quantified and analyzed by one-way ANOVA and followed by Tukey’s multiple comparison test. *p ⩽ 0.05 versus all other groups; ^#^*p* ⩽ 0.05 versus RBC only group. (c) Western blot of Mrp1 and LGN in WT mouse MG. Primary mouse MG were exposed to RBC for 24 h. The protein expression of Mrp1 and LGN was determined with Western blot. Two AhR agonists (10 µM BrB or 2 µM ITE) added to the MG together with RBC increased the LGN and Mrp1 expression. The OD of each protein band was quantified and analyzed by One-Way ANOVA and followed by Tukey’s multiple comparison test. *p ⩽ 0.05 versus all other groups; ^#^*p* < 0.05 versus RBC alone group. (d) Total BrB, (e) MTT assay, and (f) Phagocytosis Index of mouse MG with Mrp1 and/or LGN knockdown, as measured at 6 h after exposing MG to RBC. The siRNAs (10 nmol/l) were applied to the cells at 36 h before exposure to RBC. The data are mean ± SD (*n* = 5) and analyzed by one-way ANOVA, followed by Tukey’s multiple comparison test. *p ⩽ 0.05 versus control siRNA group. OD: optical density.

It is recognized that under conditions leading to the formation of hematoma, phagocytic cells conducting erythrophagocytosis accumulate high levels of BrB (Figure 1(b)). Although BrB possesses anti-oxidant properties, the accumulation of intracellular BrB could be harmful to phagocytes themselves, in part due to BrB’s extremely poor water solubility. To prevent free BrB from damaging phagocytes, these cells increased expression of the BrB-binding protein LGN and the BrB-exporting protein Mrp1 (Figure 2(c)). The LGN increases BrB’ water solubility and facilitates its transport, whereas Mrp1 acts by exporting BrB from the phagocytes to maintain the low intracellular BrB levels. We demonstrate here, using immunohistochemistry, that both LGN and Mrp1 are abundant in MG (Figure 4(a)) and that exposure of MG to RBC upregulates the MG’ expression of LGN and Mrp1, at both of mRNA (Figure 4(b)) and protein levels (Figure 4(c)). We also found that BrB and ITE, used as AhR activators, further increase MG’ LGN and Mrp1 expression (Figure 4(c)), and that AhR selective antagonist, CH-223191, reduced their expression (Figure 4(b)). This suggests the important role of AhR in regulating the expression of LGN and Mrp1 in MG. Interestingly, exposure of MG to CH-223191 reduced the engulfment of RBC by 54% as assessed at 4 h of incubation of MG with RBC (*p* < 0.05; *n* = 5).

To further establish the causal roles of LGN and Mrp1 in protecting MG for ensuring their erythrophagocytosis, we preincubated the primary MG with LGN and/or Mrp1 siRNA. Using this approach, we found an interactive effect between LGN and Mrp1 in modulating intracellular BrB (iBrB) accumulation during erythrophagocytosis. While the knockdown of LGN alone had no effect on iBrB accumulation and the knockdown of Mrp1 only modestly increased iBrB accumulation, the simultaneous knockdown of LGN and Mrp1 led to a greater accumulation of intracellular BrB (Figure 4(d)). Importantly, this greater increased iBrB accumulation was associated with the loss of MG integrity (Figure 4(e)), confirming the toxic effect of iBrB when accumulated in the phagocytes during erythrophagocytosis. This cytotoxic BrB level in MG was also associated with the loss of phagocytosis capacity (Figure 4(f)). This experiment provides important evidence that LGN and Mrp1 are important for the homeostatic modulation of the iBrB levels during erythrophagocytosis in protecting MG from fBrB’s cytotoxicity, and the ability to activate AhR, which is needed for their proper phagocytosis function.

### AhR expression is elevated in brain following ICH

**Figure 5.**
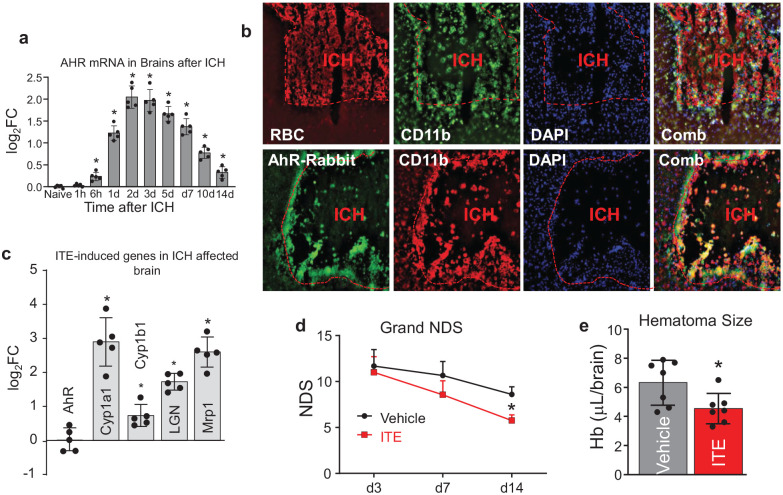
(a) Temporal profile of AhR mRNA expression in ICH-affected mouse striatal tissues (locus of blood injection) of C57/BJ6 (WT) male mice at 1 h–14 days post-ICH, as measured by qRT-PCR. The data are expressed as fold changes over the naïve mice and displayed as mean ± SD (*n* = 5), and analyzed by one-way ANOVA, followed by Tukey’s multiple comparison test. *p ⩽ 0.05 versus naïve control. (b) Double immunofluorescence of RBC (red) and CD11b (green; upper row), and of AhR (green) and CD11b (red; lower row) around hematoma at 7 days after ICH. The nuclei are labeled with DAPI (blue). A colocalization (Comb) image of RBC^+^CD11b^+^DAPI (upper row), and of AhR^+^CD11b^+^DAPI (bottom row) is shown, which demonstrates that the AhR primarily localizes in the activated microglia/macrophages around the hematoma. (c) qRT-PCR of AhR regulated genes in response to ITE in C57/BJ6 mouse brain (males) at ICH-affected striatum on day 3 after ICH. The animals received ITE (8 mg/kg) or vehicle control (5% DMSO in saline) first 30 min after ICH, then 24 h late by i.p. injection. The data are expressed as fold change of the ITE-treated over the vehicle treated control group (mean ± SD, *n* = 5). The data are analyzed by one-way ANOVA, followed by Tukey’s multiple comparison test. *p ⩽ 0.05 versus vehicle control. (d) NDS and (e) hematoma resolution after ICH in WT mice treated with ITE. The AhR agonist ITE (8 mg/kg) or vehicle control (5% DMSO in saline) was delivered i.p. at 24 h after ICH and then once a day for continuous 5 days. The NDS on days 3, 7, and 14 represent a combination score of three individual behavior tests (Postural Flexing, Foot-faults, and Corner-turning). The data are expressed as mean ± SD (*n* = 9) and analyzed by repeated two-way ANOVA. *p ⩽ 0.05 versus vehicle control group. The bar graph of hematoma Hb content in the brain on day 7 after ICH in the control and the ITE-treated mice is expressed as mean ± SD (*n* = 7) and analyzed by *t*-test. *p < 0.05.

We employed an autologous blood injection model of ICH in mice and using RT-PCR, we measured the AhR expression levels in the ICH-affected brain tissue at various timepoints (Figure 5(a)). We found that the baseline expression of AhR in the brain is negligible, and that, in response to ICH, there is a robust increase in the AhR expression over time, with the peak expression on day 2 after ICH, which continues to be elevated for ~2 weeks. Then, using immunofluorescence, we identified that the majority (90.3% ± 3.5%; *n* = 5 coronal sections from five ICH-affected brains) of CD11b immunoreactive cells in the areas adjacent to hematoma-affected brain at day 7 after ICH are also AhR^+^, suggesting that AhR expression in the ICH-affected brain is associated with the activated phagocytes (Figure 5(b)).

### AhR agonist increases the expression of the AhR target genes and promotes functional recovery after ICH in mouse

Encouraged by the *in vitro* data suggesting a stimulating role of AhR on the MG-mediated erythrophagocytosis, a process that could help in hematoma cleanup and recovery after ICH, we tested if the selective activator of AhR, ITE, improves post-ICH recovery. First, to establish biological engagement of ITE, we showed that ITE enhanced the expression of AhR target genes in the ICH-affected brain tissue (Figure 5(c)). Next, in a parallel study, we tested if ITE may reduce ICH-mediated injury. Mice were subjected to ICH and 24 h later received ITE followed by additional once a day treatment for 5 days. To assess the outcome, we measured the neurological deficits (NDS; a composite score of three tests—Postural Flexing, Foot-Faults, and Corner test; Figure 5(d)) and hematoma resolution (Figure 5(e)). ITE improved ICH functional recovery, with significant improvement in sensorimotor performance on day 14 after ICH. And also, the amount of Hb remaining in the ICH-affected brain at day 7 was significantly reduced 29.2% by ITE, suggesting that ITE improves hematoma clearance after ICH.

### MG-specific AhR-KO in mice worsens post-ICH recovery and shows less benefit from AhR-based therapy

**Figure 6.**
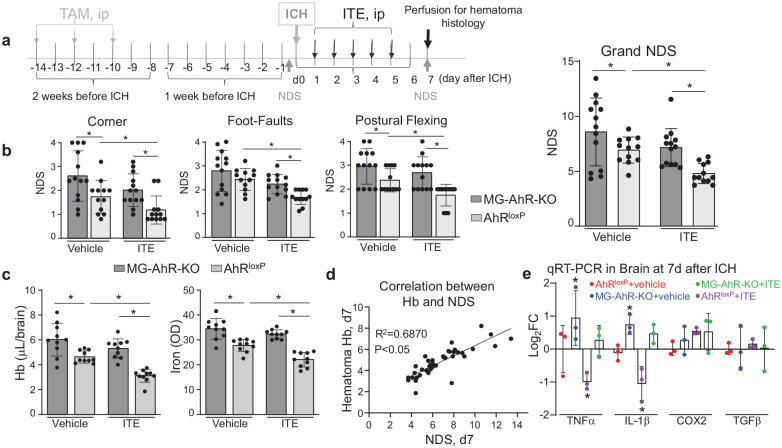
MG-AhR-KO mice have more severe neurological deficit and impaired hematoma resolution after ICH. (a) Schematic graph of the experimental design with MG-AhR-KO mice. TAM (Sigma, T5648) was i.p. injected into MG-AhR-KO and AhR^loxP^ mice at 3 mg/mouse on day 1 and at 1 mg/mouse on days 3 and 5. Nine days later the mice were subjected to ICH. ITE was delivered i.p. at 8 mg/kg, 24 h after induction of ICH, then once a day for 5 days. (b) The neurological deficit (NDS) was measured by Footfault, Postural Flexing, and Corner Turning tests, and by the composite of all the three tests (Grand NDS) on day 7 after ICH-induced injury. The data are mean ± SD (*n* = 12/group). (c) The residual hematoma on day 7 after ICH as measured by quantitation of remaining hemoglobin and iron in the ICH-affected brain. The data are mean ± SD (*n* = 10/group). The data in (b) and (c) are analyzed by repeated two-way ANOVA. *p < 0.05. (d) Correlation between Hb remaining in the brain at 7 days after ICH and neurological deficit (Grand NDS) on the same day, demonstrating that less hematoma remained in the brain better neurological recovery took place. *N* = 39, Pearson correlation coefficient *R*^2^ = 0.69, *p* < 0.05. (e) Bar graph of RT-qPCR data in MG-AhR-KO and AhR^loxP^ mice brain after ICH and ITE treatment. The inflammatory genes expression (TNFα, IL-1β, COX2, and TGFβ) at 7 days after ICH in ICH affected brain subcortex of male mice were quantified by RT-qPCR. The data are first normalized by the 18 s used as internal control, then compared to the AhR^loxP^ vehicle group, and expressed as Log_2_FC. The data are mean ± SD (*n* = 3). The data were analyzed by one-way ANOVA, followed by Tukey’s multiple comparison test. *p ⩽ 0.05 from AhR^loxP^ vehicle. TAM: tamoxifen.

To further clarify the role of AhR in MG in post-ICH recovery, we next employed inducible MG-specific AhR knockout mice (MG-AhR-KO) generated in our animal facility. The experimental design of this experiment is described in Figure 6(a). Briefly, 2 weeks before the induction of ICH all mice (including control) received tamoxifen to trigger Cre-mediated knockout. AhR^loxP^ mice in the same litters with the same age and matched sex were used as the control. ICH was induced using autologous blood injection model.^4^ Twenty-four hours after ICH, then once a day for a total of five consecutive days, animals received intraperitoneally ITE (8 mg/kg) or vehicle. The outcome (neurological deficits, hematoma resolution) was measured on day 7 after ICH. Using this approach, we established that in response to ICH, MG-AhR-KO mice, as compared to AhR^loxP^ control mice, showed worse neurological performance including on Postural Flexing test and Corner test (Figure 6(b)), with the grand NDS (a combination score of all three behavioral tests) showing 24% worse performance, than the control group. This indicates that the deficiency of AhR in MG is detrimental to post-stroke functional recovery. Analysis of control animals (AhR^loxP^) treated with ITE confirms our conclusions produced in wild-type mice (Figure 5(d)), demonstrating that ITE significantly improved post-ICH recovery (Figure 6(b)). In contrast to the beneficial role of ITE in AhR^loxP^ mice, ITE in MG-AhR-KO mice showed trends, but not significant benefits. This further implicates the important role of activation of AhR, specifically in MG, in the recovery after ICH. When comparing sex differences, the NDS in males trended to be more severe, 10.3% higher in MG-AhR-KO mice (*p* = 0.1; *n* = 12/groups) and 5.7% higher in AhR^loxP^ control mice (*p* = 0.47; *n* = 12/group). Essential to our hypothesis, the degree of hematoma resolution (measured by quantitating Hb and iron remaining in the brain at day 7) was significantly improved with ITE treatment in the AhR^loxP^ control mice, but not in MG-AhR-KO mice (Figure 6(c)). Hematoma resolution in females trended to be slightly faster (9.8% faster in MG-AhR-KO mice, *p* = 0.22 and 8.4% faster in AhR^loxP^ control mice, *p* = 0.41; *n* = 12/group). These trends were similar for AhR-KO and the control groups. Our findings suggest that one of the potential mechanisms leading to improved post-stroke recovery by AhR is achieved by improved hematoma resolution, suggesting that AhR-activated MG-mediated clearance of hematoma could be the mechanism of improved recovery. To further claim this assertion, we are demonstrating, using analysis correlating behavioral performance (Grand NDS) with the amount of brains’ remaining Hb (index of hematoma resolution) at day 7 after stroke for mice used in this experiment that hematoma resolution positively correlated with the behavioral recovery (Figure 6(d)). Finally, we observed a reduction in mRNA expression of TNFα and IL-1β, and no effect on expression of COX2 and TGFβ in ICH-containing tissue in control mice treated with ITE but not in MG-AhR-KO mice (Figure 6(e)).

## Discussion

The main hypothesis of this study was that the activation of AhR in MMΦ after ICH represents a novel therapeutic target for post-ICH recovery, based on AhR’ ability to improve MMΦ self-defense from toxic byproducts of erythrophagocytosis, thereby leading to more effective hematoma cleanup, which is needed for brain repair and functional recovery.

Using in vitro ICH-like condition (exposing MG to RBC), we found that the activation of AhR in MG plays an important role in promoting erythrophagocytosis by enhancing the expression of BrB handling protein LGN and Mrp1 to prevent intracellular accumulation of cytotoxic levels of fBrB. Using a translationally relevant model of ICH, we then showed that mice with selective AhR deficiency in MG have worse outcomes (neurological deficit and hematoma resolution) than the control mice, and systemic treatment of mice after ICH with a selective AhR agonist (ITE) improved the recovery in the control (loxP) mice, but not in mice with genetic deficiency of AhR, selectively in MG. Interestingly, though not statistically different, a trend toward the reduction of NDS by ITE in MG-AhR-KO mice was noted. Although entirely speculative, the residual AhR activity in MG-AhR deficient mice due to incomplete AhR knockout in MG and/or the recruited blood derived macrophages could contribute to these ITE pharmacologic effects. Overall, the results strongly suggest that AhR-mediated signaling in MG is essential for post-ICH recovery.

It is generally accepted that after ICH, RBCs, the main component of cerebral hematoma, undergo progressive lysis, leading to generation of masses of highly cytotoxic hemoglobin, heme, and iron. Thus, hematoma is a reservoir of toxicity and pro-inflammatory responses that continue to harm the surrounding brain for weeks or months and impede repair.^54–56^ To combat this pathology the MMΦ surrounding hematoma conduct phagocytosis/endocytosis-mediated cleanup of the hematoma debris. Over the years, several molecular pathways known to control MMΦ-mediated phagocytosis, for example, CD36,^4^ LRP1,^57^ AXL/MERTK,^58^ CD47^59^ were demonstrated to help in hematoma clearance and improved recovery in animal models of ICH. This demonstrates that targeting MMΦ for effective hematoma resolution may represent a promising therapeutic strategy for ICH.

During erythrophagocytosis, MMΦ that internalize high loads of RBC/Hb build high intracellular levels of heme.^60^ Heme is highly cytotoxic, acts as pro-oxidant, and induces MΦ senescence,^60,61^ processes that are detrimental to phagocytic and “reparative/trophic” functions of the phagocyte.^7,55^ To eliminate the rapidly accumulating heme, MMΦ upregulate the heme degrading heme oxygenase-1 (HO-1), a rate limiting enzyme that converts heme to iron, CO, and biliverdin, which is then converted to free bilirubin (fBrB).^7–9,60^ In agreement with this sequalae, we observed a robust increase in BrB levels in MG during their erythrophagocytosis, and this BrB accumulation is prevented by HO-1 inhibitor, SnPP. BrB itself possesses anti-oxidative properties.^62^ However, fBrB is almost insoluble in water^10,11^ and without the presence of its binding proteins, fBrB aggregates at concentration as low as 1 μM or even 140 nM.^10,12–14^ The overproduction and accumulation of fBrB could lead to the formation of metastable microsuspensions and coarser aggregates, which precipitate on biological membranes and nucleic acid, causing structural and oxidative damage to intracellular organelles and DNA.^10,11,15^ Thus, despite its beneficial anti-oxidative properties,^62^ the buildup of supraphysiological levels of fBrB in the MMΦ during erythrophagocytosis could be toxic to the phagocytes and cause their dysfunction and injury.

To keep fBrB soluble the cells produce specialized factors that directly bind fBrB to maintain its solubility, such as LGN, as well as factors that control the intracellular level of BrB by facilitating its efflux, such as Mrp1. LGN is a 47 kDa heterodimer, which is identical to enzyme glutathione-S-transferases (GSTs).^27^ GSTs are a super-family of phase II metabolizing enzymes that catalyze the detoxification of a large range of endogenous and exogenous toxic compounds, playing an important role in protecting cells against damage, including through glutathione conjugation with electrophilic substances.^63,64^ LGN binds hydrophobic fBrB to increase its water solubility^17–21^ and it acts in the cytosol as an intracellular transporter,^22–24^ as does albumin in blood plasma.^25–27^ The A subunit (GSTya) of LGN contains a single high-affinity binding domain for BrB with an association constant estimated as 5 × l0^7^–7 × l0^6^ M-1.^20^ Normally, LGN expression is regulated through transcription factor, aryl hydrocarbon receptor (AhR).^16,34,35^

While LGN is assisting in fBrB solubility, the intracellular level of BrB is controlled through its efflux from the cell, mediated through BrB exporting proteins including Mrp1,^28,29^ which pumps fBrB out of the cells to prevent formation of BrB aggregates. Mrp1 is the first identified member of the Mrp subfamily of ATP-binding-cassette transporters that supports BrB efflux.^65^ Mrp1 binds fBrB with the highest affinity (Km for fBrB = 10 nM) of any substrate so far reported, which allows to export fBrB with high specificity.^29,30^ Once exported and in the blood stream, fBrB binds albumin (referred to as unconjugated BrB)^66–68^ and is transported to hepatocytes for glucuronic acid conjugation, and removal via bile system.^69^ It is important to emphasize that similar to LGN, Mrp1 expression, is also regulated by AhR.^70^ In agreement with the important role of LGN and Mrp1 in supporting phagocyte’s proper activity, we now demonstrated that knockdown of both LGN and Mrp1 leads to increased accumulation of BrB in MG during erythrophagocytosis that coincide with impaired MG’ phagocytic function and injury to MG. Thus, knowing that LGN and Mrp1 are important for health of MG and optimal erythrophagocytosis and that AhR can amplify the expression of both LGN and Mrp1, we further evaluated the role of AhR in ICH.

AhR is a ligand-dependent transcription factor that binds, as a dimer with ARNT (AhR nuclear translocator), to xenobiotic response element sequences in target gene promoters to regulate the expression of genes essential for metabolism/detoxification of pollutants, xenobiotics, and some endogenously produced metabolites.^36–39,71,72^ Intriguingly, BrB is an endogenous AhR activator.^39,40^ In agreement with that role, phagocytosis of RBC (process resulting in a robust increase in BrB production) causes activation of AhR in MG and transcription of prototypic AhR genes, including Cyp1a, as well as LGN and Mrp1, whereas, phagocytosis of RBC by AhR deficient MG led to reduced production of Cyp1a, LGN, and Mrp1. To single out BrB in MG as an important factor regulating erythrophagocytosis, and therefore hematoma cleanup through AhR activation, we demonstrated that while BrB enhances erythrophagocytosis in control MG, BrB does not affect erythrophagocytosis in MG harvested from MG-selective AhR knockout mice. Overall, based on our in vitro studies, we assumed that the activation of AhR under ICH-like environment improves MG’s health and phagocytic function by limiting the excessive accumulation of BrB through upregulation of LGN and Mrp1.

Our ultimate goal was to probe if AhR, that is, abundant in MG acts as a regulator of phagocytic function to benefit post-ICH hematoma clearance and functional recovery. To test this hypothesis, we generated MG-selective (TMEM119-Cre driven recombination in AhR^loxP^ mice) AhR-knockout mice and then subjected them to ICH. These animals appeared to be normal based on their weight, activity and appearance. However, when subjected to ICH, MG-AhR-KO mice showed greater neurological deficit, as compared to the AhR^loxP^ control. They also experienced defective brain cleanup, as established by demonstrating that the amount of blood (specifically, Hb and iron) left in the brain at day 7 after ICH was greater in AhR-KO mice than in the control mice. These results are in agreement with our and other’s earlier finding that the loss in MMΦ’ ability to conduct efficient cleanup, for example, due to MMΦ-selective deficiency of CD36, CD91, RXR, or AXL/MERTK renders worse recovery after ICH-induced injury.^4,6,55,57,58^ To further define AhR in MG as target for post-ICH recovery, we treated AhR-KO and loxP control animals with AhR agonist. Since, use of BrB as AhR ligand is impractical due to its poor solubility and delivery to the intracellular compartment, we used another natural selective AhR ligand, tryptophane derivative, ITE.^73^ As anticipated, we established that ITE treatment initialed as late as 24 h after ICH (to preferentially target mechanism associated with recovery phase) was effective in reducing functional deficits and in improving brain cleanup in control animals, but not in the MG-AhR-KO mice.

The role of AhR in MMΦ as positive regulator in phagocytosis of RBC may not be universal. While we demonstrated that phagocytosis of RBC by microglia is enhanced by BrB and ITE (AhR ligands) and that AhR deficiency in microglia negatively impacted phagocytosis, Zahringer et al. when studying acute graft-versus-host disease observed that microglia in culture showed deficient phagocytosis toward microbeads, upon exposure to AhR ligand 6-formylindolo(3,2-b)carbazole.^74^ This discrepancy could be related to the use of different AhR ligands or phagocytic targets.

Overall, we found that BrB is elevated in MG upon phagocytosis of RBC, which could activate AhR and upregulate expression of Mrp1 and LGN, contributing to MG’ self-protection for more efficient phagocytosis function; and the AhR agonists could promote hematoma resolution and benefit post-ICH recovery in an animal model of ICH.

## Supplemental Material

sj-pptx-1-jcb-10.1177_0271678X251371375 – Supplemental material for Neurological recovery after ICH is mediated by the aryl hydrocarbon receptor-bilirubin interplay through improved erythrophagocytosis Supplemental material, sj-pptx-1-jcb-10.1177_0271678X251371375 for Neurological recovery after ICH is mediated by the aryl hydrocarbon receptor-bilirubin interplay through improved erythrophagocytosis by Xiurong Zhao, Shun-Ming Ting, Guanghua Sun and Jaroslaw Aronowski in Journal of Cerebral Blood Flow & Metabolism
