# Supplementary material for: Neurological recovery after ICH is mediated by the aryl hydrocarbon receptor-bilirubin interplay through improved erythrophagocytosis
Source: J Cereb Blood Flow Metab. 2025 Sep 17:0271678X251371375. Online ahead of print. doi: 10.1177/0271678X251371375 (PMC12446277; doi:10.1177/0271678X251371375)
Supplement: sj-pptx-1-jcb-10.1177_0271678X251371375 – Supplemental material for Neurological recovery after ICH is mediated by the aryl hydrocarbon receptor-bilirubin interplay through improved erythrophagocytosis [file sj-pptx-1-jcb-10.1177_0271678X251371375.pptx]

## Slide 1
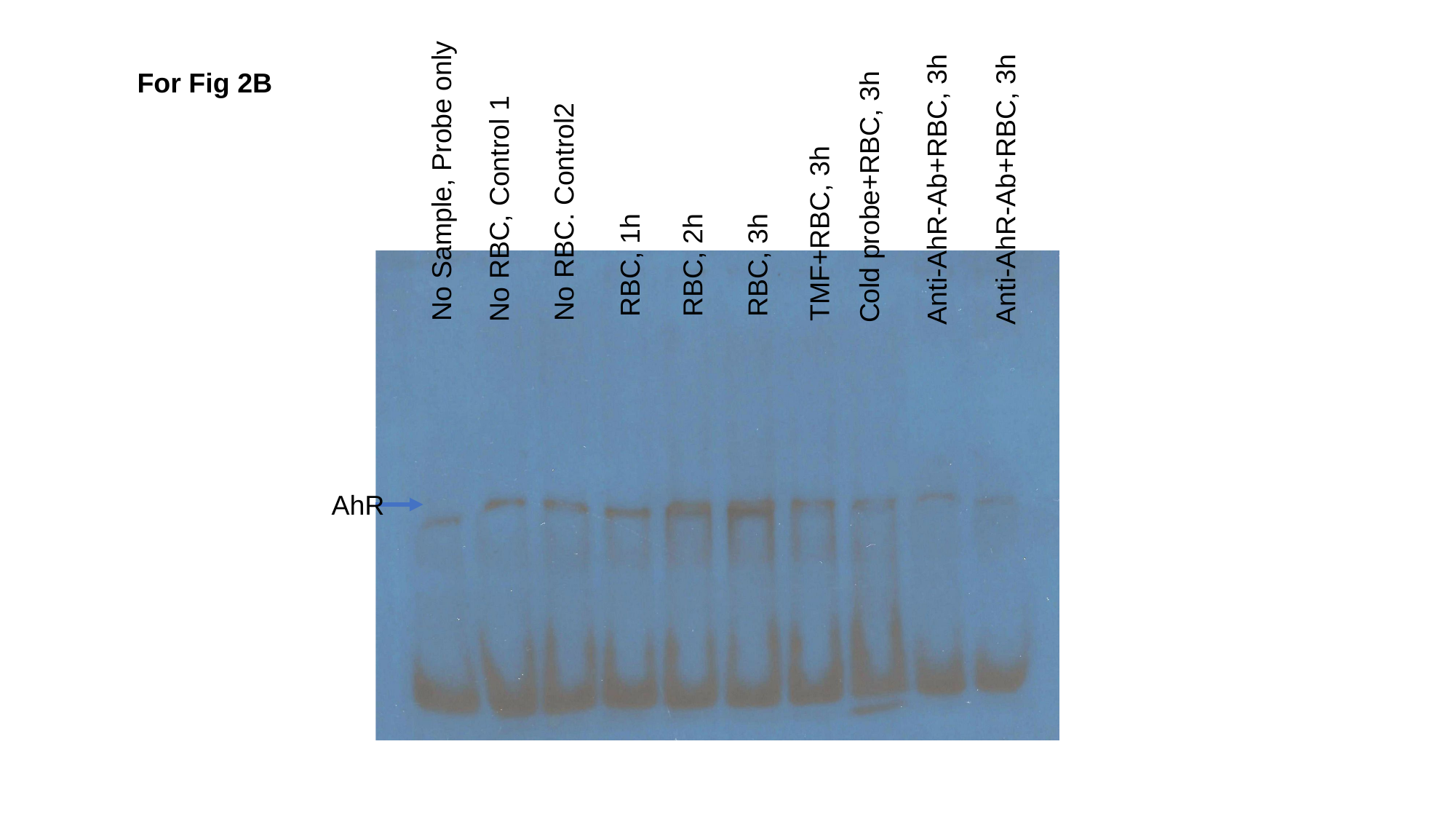

For Fig 2B
No Sample, Probe only
Anti-AhR-Ab+RBC, 3h
Anti-AhR-Ab+RBC, 3h
Cold probe+RBC, 3h
No RBC, Control 1
No RBC. Control2
TMF+RBC, 3h
RBC, 1h
RBC, 2h
RBC, 3h
AhR

## Slide 2
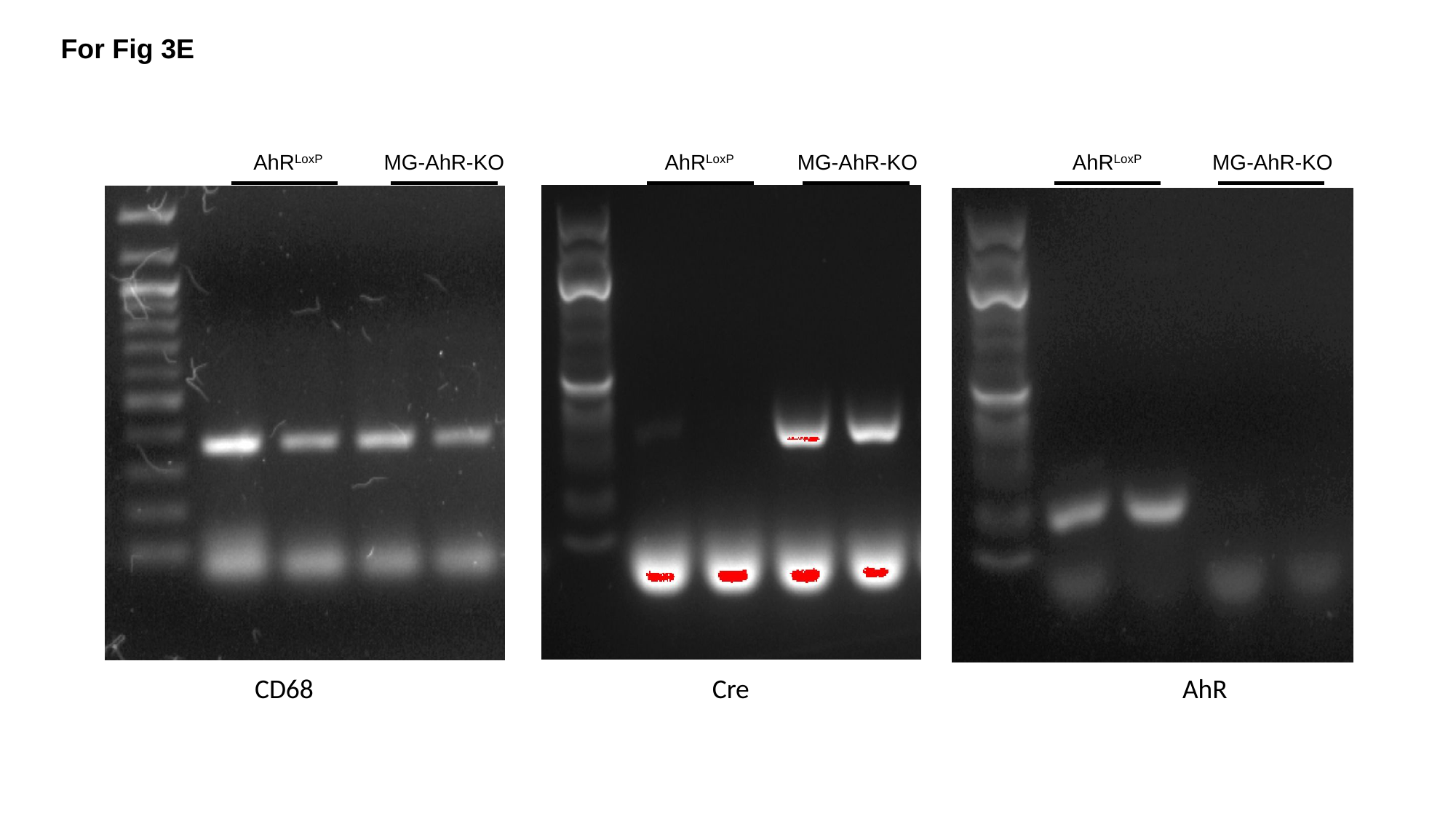

For Fig 3E
AhRLoxP
MG-AhR-KO
AhRLoxP
MG-AhR-KO
AhRLoxP
MG-AhR-KO
CD68
Cre
AhR

## Slide 3
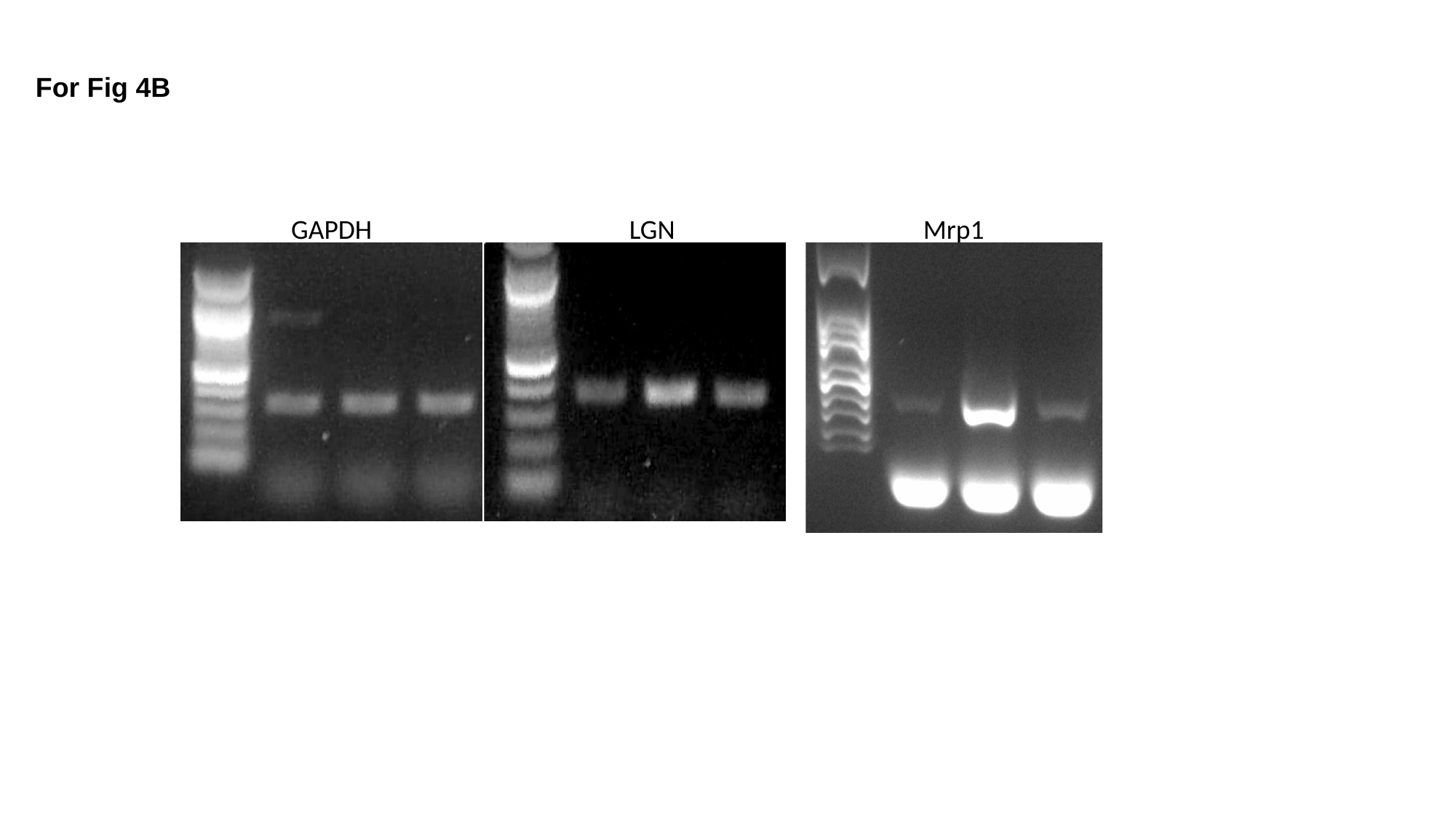

For Fig 4B
GAPDH
LGN
Mrp1

## Slide 4
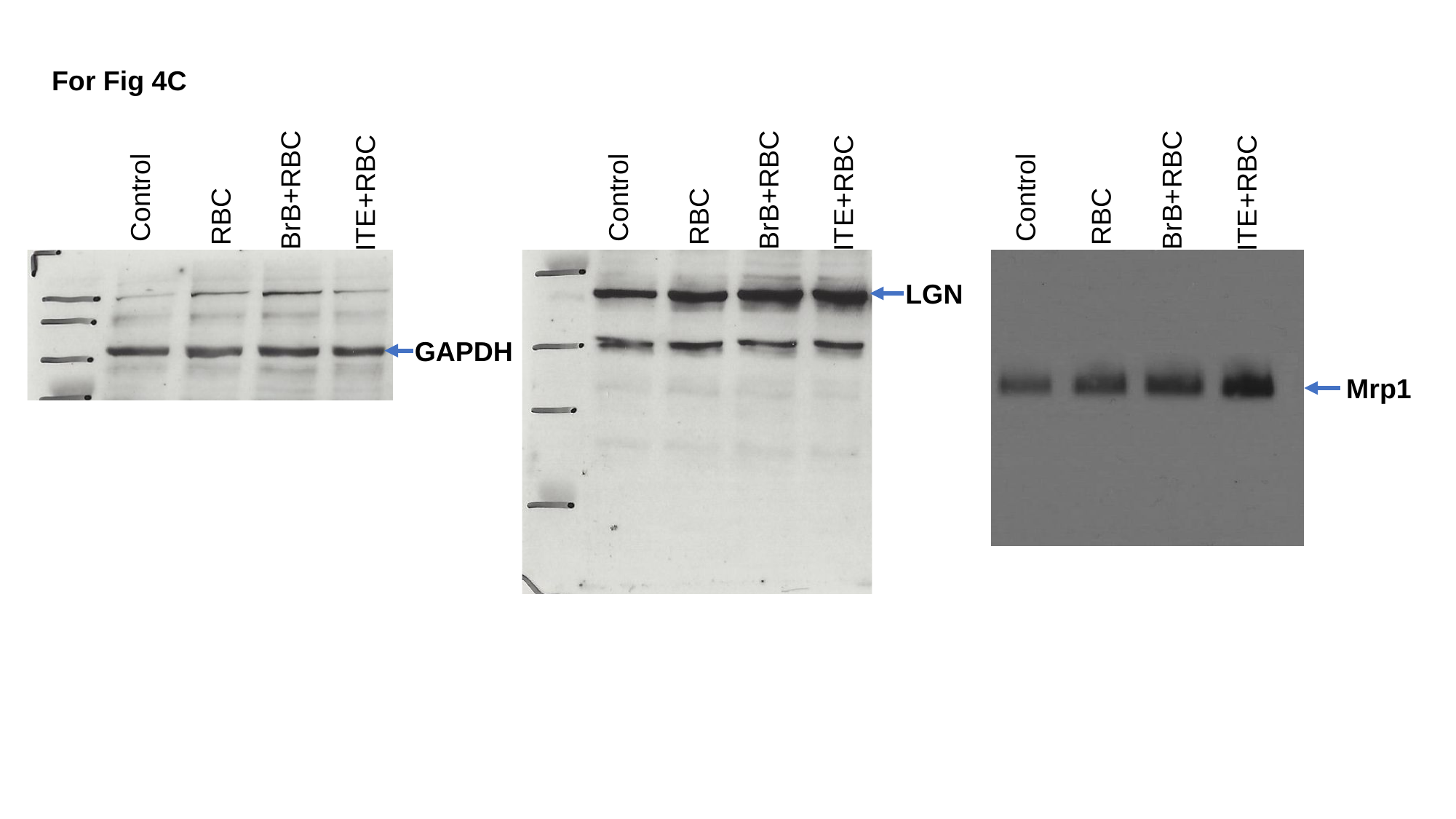

For Fig 4C
BrB+RBC
BrB+RBC
BrB+RBC
ITE+RBC
ITE+RBC
ITE+RBC
Control
Control
Control
RBC
RBC
RBC
LGN
GAPDH
Mrp1
